# Impact of pressure ulcers and frailty on long-term mortality: a prospective cohort study of hospitalized older adults

**DOI:** 10.1007/s41999-025-01331-8

**Published:** 2025-11-14

**Authors:** Silvia Ottaviani, Eleonora Rondanina, Elena Longo, Floriana Arnone, Virna Brucato, Roberto Campigli, Massimo Della Bona, Luca Tagliafico, Stefania Peruzzo, Alessio Nencioni, Fiammetta Monacelli

**Affiliations:** 1 Section of Geriatrics, Department of Internal Medicine and Medical Specialties (DIMI), University of Genoa, Viale Benedetto XV, 6, 16132 Genoa, Italy; 2 IRCCS Ospedale Policlinico San Martino, Genoa, Italy; 3 ASL5 Ospedale San Bartolomeo, Sarzana, Italy

**Keywords:** Pressure sores, Immobility, Frailty, Mortality, Comprehensive geriatric assessment

## Abstract

**Purpose:**

This study investigates the impact of pressure ulcers (PU) on long-term survival in hospitalized older adults, to understand how frailty and PU interact to influence mortality and to explore their independent prognostic accuracy.

**Methods:**

Between February 21, 2022, and July 1, 2022, 324 patients (198 female, 126 male; mean age: 86), admitted to the Geriatric Unit and Transitional Care of San Bartolomeo Hospital, Sarzana, Italy, were enrolled. A comprehensive geriatric assessment (CGA) was performed by geriatricians, including the assessment of frailty using the Clinical Frailty Scale (CFS), and PU assessment, carried out by a trained nurse. Long-term mortality (median duration: 18 months) was collected through the regional electronic health data system with an 18-month follow-up period.

**Results:**

Older adults with PU had advanced frailty, higher nutritional risk, increased disability, and higher polypharmacy. Multivariate analysis showed that both frailty (CFS: HR 1.535, 95% CI 1.314–1.793, *p* < 0.001) and PU (HR 1.500, 95% CI 1.059–2.126, *p* = 0.020) were significant predictors of long-term mortality. Namely, each increment in CFS score was associated with a 53% increased risk in PU carriers. No significant interaction between CFS and PU on survival outcomes was found.

**Conclusion:**

The study underscored the independent prognostic significance of both PU and frailty in predicting long-term mortality in hospitalized older adults. A multidimensional approach considering both frailty and PU is crucial for accurate prognostication and improving care strategies for older patients.

**Supplementary Information:**

The online version contains supplementary material available at 10.1007/s41999-025-01331-8.

## Key summary points

**Aim:**

To investigate the independent and combined impact of pressure ulcers (PU) and frailty on long-term survival in hospitalized older adults.

**Findings:**

Both PU and frailty were independently associated with increased long-term mortality. Each one-point increase in the Clinical Frailty Scale was linked to a 53% higher mortality risk, and the presence of PU added an additional 50% risk, regardless of age, sex, and comorbidity. No significant interaction between frailty and PU was observed.

**Message:**

Assessing both frailty and pressure ulcers is essential for accurate long-term prognostication in hospitalized older patients.

**Supplementary Information:**

The online version contains supplementary material available at 10.1007/s41999-025-01331-8.

## Introduction

Despite advancements in the development of effective prevention and treatment approaches, pressure ulcers (PU) persist as a prevalent and burdensome clinical condition, impacting healthcare systems and socioeconomic costs, especially in older adults. Data from the Global Burden of Disease Study 2019 underscored that the age-standardized prevalence rate of PU is approximately 11.3, with an incidence of 41.8, and 1.7 years lived with disability per 100,000 population [1, 2]. PU are associated with increased mortality, prolonged hospital stays, a higher risk of infection, in-hospital malnutrition, and an increased need for nursing care, all contributing to outgrowing healthcare costs [3, 4].

Several studies have highlighted the prognostic implications of PU [5]. For instance, it was observed that older adults who developed in-hospital PU were more likely to experience increased 30-day mortality [6]. Similarly, older adults with PU demonstrated reduced survival rates, showing an unfavorable interaction between PU and dementia on the determination of long-term survival [7]. In orthogeriatric patients with hip fracture, the development of PU was associated with higher 6-month mortality [8].

Recently, the impact of physicians’ clinical judgment on the management of multimorbid older adults with PU emphasized the role of the assessment of frailty as a critical component [9]. Frailty is characterized by increased vulnerability to physiological or psychological stressors, and it is associated with higher rates of adverse outcomes, including mortality, comorbidity, and functional decline [10]. The Clinical Frailty Scale (CFS) has emerged as a valuable tool for the stratification of frailty [11], indicating its ability to predict survival outcomes in different old-age populations [11–13], however, its application in the specific context of PU remains limited. Previous studies, like that of Donini et al. [14], highlight how higher frailty levels worsen PU progression, yet the lack of systematic integration of frailty assessment in PU management contributes to a poor understanding of their interplay, hindering the identification of high-risk patients. Moreover, the recent systematic review by Alotaibi et al. underlines the role of CFS as a predictor of hospital-related adverse events, including PU development [15]. It remains unclear whether PU and frailty should be regarded as independent predictors of mortality or whether their coexistence exerts a synergistic effect that accelerates poor outcomes. While both conditions are established risk factors for mortality, the available literature has examined them mostly in isolation, and no studies have explored their combined prognostic impact using validated instruments such as the CFS.

Building upon this background, the present prospective observational study aims to evaluate the impact of PU on the long-term survival of a cohort of hospitalized old-age individuals while accounting for the influence of frailty, exploring whether their coexistence exerts a synergistic effect, potentially accelerating mortality risk beyond the impact of each condition alone.

## Methods

During a 5-month time (between 21/02/2022 and 01/07/2022), 382 patients were admitted to the Geriatric Unit and Transitional Care of San Bartolomeo Hospital in Sarzana (La Spezia, Italy). This acute care ward primarily admits patients, aged ≥ 65 years, directly from the Emergency Department or from the medical intensive care area, with a mean length of stay of approximately 5 days.

Within 48 h after hospital admission, a Comprehensive Geriatric Assessment (CGA) was conducted by an expert geriatrician, including the risk of falls (Conley scale)[16] and delirium (4AT)[17]; functional status (Barthel and IADL)[18, 19]; multimorbidity (Cumulative Illness Rating Scale CIRS) [20]; cognitive status (Short Portable Mental Status Questionnaire SPMSQ)[21]; nutritional status (Mini Nutritional Assessment MNA)[22]. The number of medications at hospital admission and at discharge and the Anti-Cholinergic Burden (ACB)[23] were also collected. To assess sarcopenia, Hand Grip (Kuptone dynamometer, model: EH101), mid-arm circumference (MAC), mid-thigh circumference (MTC), and triceps skin fold (TSF) were collected. Frailty status was assessed with the Clinical Frailty Scale (CFS)[11], following the same procedures applied to the other components of the CGA.

Additionally, PU assessment was performed by a trained geriatric nurse at the time of hospital admission through direct clinical examination; PU were staged according to NPUAP/EPUAP classification [24]. Patients’ risk to develop PU was stratified according to the Braden scale [25].

Long-term mortality was collected through the regional portal (ASL5 La Spezia, Italy electronic health data), considering the censorship criteria (censorship date: 1 st September 2023) and the duration of follow-up (median duration 18 months). No missing data were observed for the outcomes.

Inclusion criteria were age ≥ 65 years and provision of informed consent by the patient or a legal representative. Exclusion criteria included refusal to participate, inability to undergo the CGA due to hemodynamic instability or terminal illness, and length of stay insufficient to complete the assessment. All eligible patients were consecutively identified and approached for enrollment by the study investigators (SO and ER). Both investigators screened for inclusion/exclusion criteria, obtained informed consent, and ensured that the Comprehensive Geriatric Assessment was completed within 48 h of admission.

This study was approved by the Clinical Research Ethics Committee of the University of Genoa (No: 2024.54) and it was performed in accordance with the ethical standards of the Declaration of Helsinki.

### Statistical analysis

Bivariate testing, including chi-square tests for categorical variables and the Mann–Whitney test for continuous variables, was used to compare baseline clinical variables between patients with and without PU. The association of PU and other candidate predictor variables with survival from the date of discharge until the observation date was evaluated using the log-rank test. Unadjusted hazard ratios for each variable were derived via Cox proportional hazards regression. The survival curves for each group analysis were generated, supporting the assumption of proportional hazards for these data. A multivariable Cox regression model was then conducted to assess the independent contribution of frailty and PU to long-term mortality. Variables were selected based on significance in the univariable model and collinearity (through Spearman’s correlation test); age, sex, and comorbidity were considered potential confounders, included in the model to adjust for their possible influence on the outcome. To explore the interaction between CFS and PU on survival, the interaction term was also tested.

Importantly, although recent updates define CFS = 4, previously “*vulnerable*”, as “*very mild frailty*”[26], in our study we defined frailty as CFS ≥ 5 for descriptive purposes and for generating Kaplan–Meier survival curves. This cut-off was chosen because it is still widely used in the literature and because the number of patients with CFS = 4 was too limited to allow stable estimates [27]. Importantly, in Cox regression analyses, the CFS was analyzed as a continuous variable, thus avoiding information loss due to categorization.

All reported analyses were performed using RStudio (Version 2022.07), with a two-sided α level less than 0.05 considered statistically significant.

## Results

**Fig. 1:**
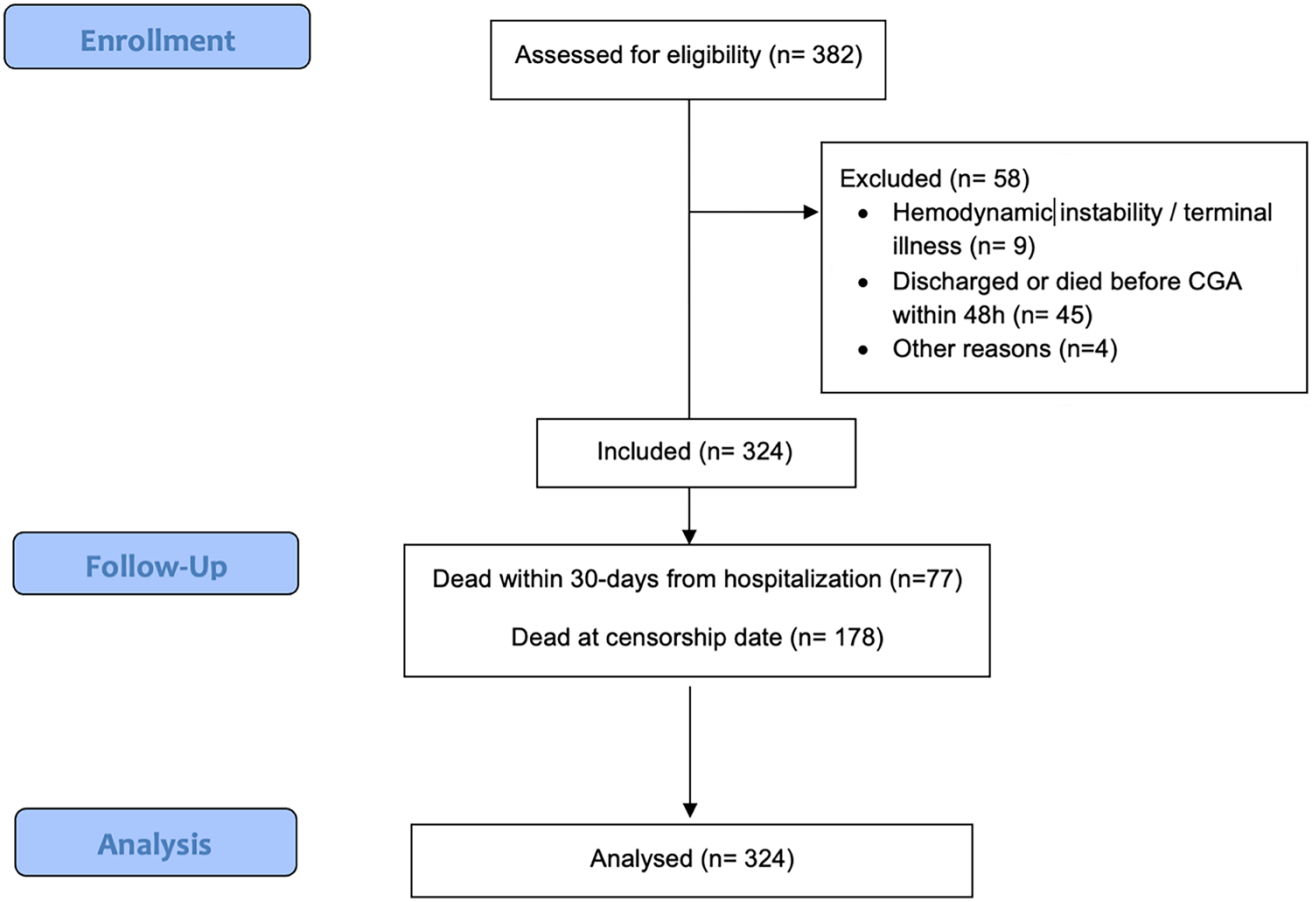
Flowchart of the study

324 patients (198 female and 126 male) were continuously enrolled in the study (see Fig. 1).

At baseline, patients’ mean age was 86 ± 10.5 years (ranging from 64 to 101 years), with 56% being oldest-old (aged 85 or older). PU carriers showed advanced frailty status (median CFS 7, IQR 0), increased nutritional risk (MNA 13, IQR 9), higher disability (Barthel index 15, IQR 41), higher incident delirium (4AT 5/12, IQR 6), and polypharmacy (average number of drugs: 6, IQR 3) (Table 1). Out of a total of 324 patients, 152 presented with PU. The analysis of these cases revealed that 60% were classified as Stage 1, followed by 26% in Stage 2. Stage 3 accounted for 7% of the cases, while 4% were categorized as Stage 4. Additionally, 3% of the PUs were deemed unstageable.

**Table 1:** Clinical phenotype of the study population as for PU presence

	PU−(*N* = 172, 53%)	PU + (*N* = 152, 47%)	*p*
Male sex, num (%)	80 (25)	46 (14)	0.003
Age, median (IQR)	85 (10)	87 (9)	0.002
Triceps skin fold, median (IQR)	16.5 (11)	16 (10)	0.258
F	18 (9.75)	16 (10)	
M	14 (10)	16 (12)	
Mid-arm circumference, median (IQR)	26 (5.6)	24 (6)	< 0.001
F	25.7 (5.4)	23 (6)	
M	26.2 (5.2)	25 (5)	
Mid-thigh circumference, median (IQR)	31 (6.5)	29 (5.5)	< 0.001
F	31 (6.5)	29 (5)	
M	32 (5.5)	30.3 (6.5)	
Hand Grip, median (IQR)	11 (7.6)	5.9 (4.9)	< 0.001
F	8.5 (5.5)	5.4 (4.1)	
M	11.6 (9.7)	9.1 (6.0)	
Conley’s scale, median (IQR)	3 (3)	5 (3)	< 0.001
IADL, median (IQR)	3 (5)	0 (1.5)	< 0.001
Barthel Index, median (IQR)	55 (55)	15 (40)	< 0.001
CIRS-comorbidity index, median (IQR)	5 (3)	5 (3)	0.140
CIRS-severity index, median (IQR)	2.15 (0.54)	2.23 (0.71)	0.205
SPMSQ, median (IQR)	6 (4)	3 (5)	< 0.001
MNA, median (IQR)	20 (8)	13.5 (10)	< 0.001
ACB score, median (IQR)	1 (2)	1 (2)	0.038
No. medication pre-hospitalization, median (IQR)	6 (4.25)	6 (4)	0.689
4AT, median (IQR)	1 (5)	5 (6)	< 0.001
CFS, median (IQR)	6 (3)	7 (0)	< 0.001

*IADL* instrumental activities of daily living, *CIRS* cumulative illness rating scale, *SPMSQ* short portable mental status questionnaire, *MNA* Mini Nutritional Assessment, *ACB* anti-cholinergic burden, *CFS* clinical frailty scale

A comparative table of frail and non-frail patients, including PU prevalence, is available in Supplementary Material S1.

Namely, 24% (77/324) of the patients died during hospitalization or within 30 days from discharge. The overall long-term mortality was 55% (178/324) which raised to 71% (108/152 patients) in patients with PU.

The multivariate model adjusted for age and sex showed that CFS, with an HR of 1.535 (95% CI 1.314–1.793, *p* < 0.001), and the presence of PU, with an HR of 1.500 (95% CI 1.059–2.126, *p* = 0.020), as well as the CIRS Comorbidity Index, were the main determinants of long-term mortality (Harrell’s concordance index of 71%, SE = 0.021).

**Fig. 2:**
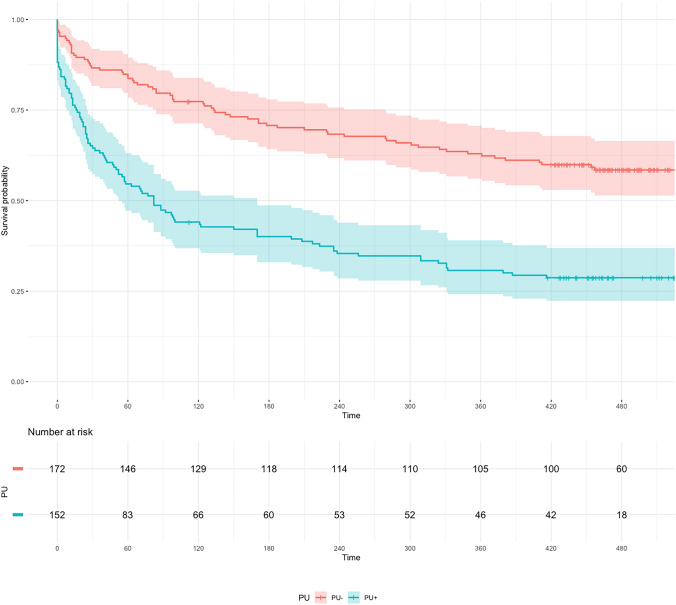
Kaplan–Meier curve stratified by presence/absence of PU

**Fig. 3:**
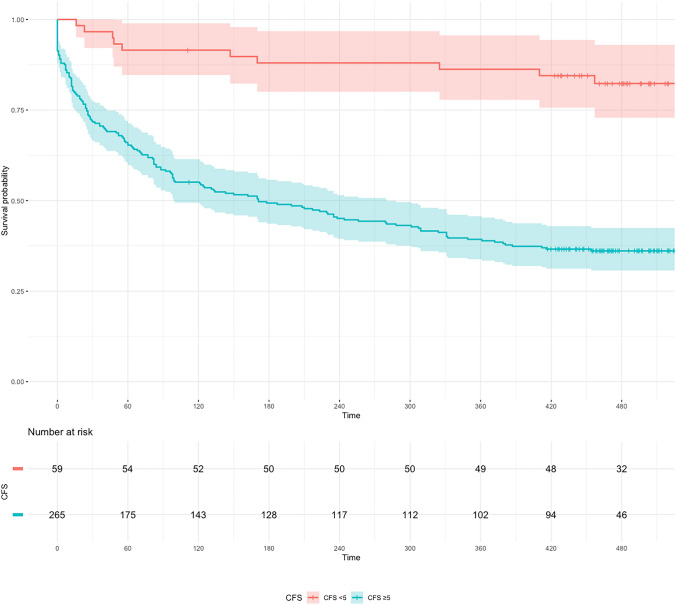
Kaplan–Meier curve stratified by frailty status (Clinical Frailty State ≥ 5)

Kaplan–Meier curves showed a significant increase in long-term mortality when stratified for the presence of frailty and PU (Figs. 2 and 3).

The interaction term between CFS and PU did not show significance (*p* = 0.897) on survival outcomes in our population. Collinearity between CFS and PU was formally assessed using Spearman’s test, which only showed a moderate correlation (ρ = 0.43, *p* < 0.001) (Table 2).

**Table 2:** Univariate and multivariate survival analysis

	Univariate analysis	Multivariate analysis
	HR (95% CI) *p* value	HR (95% CI) *p* value
Triceps skin fold	0.968 (0.948–0.990) *p* = 0.003	
Mid-arm circumference	0.925 (0.894–0.958) *p* < 0.001	
Mid-thigh circumference	0.901 (0.867–0.936) *p* < 0.001	0.963 (0.922–1.006) *p* = 0.081
Hand grip	0.957 (0.922–0.994) *p* = 0.020	
Conley scale	1.107 (1.038–1.181) *p* = 0.001	
IADL	0.785 (0.729–0.846) *p* < 0.001	
Barthel	0.980 (0.975–0.985) *p* < 0.001	
CIRS Comorbidity Index	1.057 (1.032–1.083) *p* < 0.001	1.036 (1.011–1.061) *p* = 0.004
CIRS Severity Index	1.173 (1.095–1.257) *p* < 0.001	
SPMSQ	0.835 (0.795–0.876) *p* < 0.001	
MNA	0.906 (0.884–0.928) *p* < 0.001	
ACB score	1.022 (0.927–1.126) *p* = 0.661	
Polypharmacy	1.027 (0.973–1.083) *p* = 0.325	
4AT	1.129 (1.088–1.172) *p* < 0.001	1.043 (0.993–1.095) *p* = 0.088
CFS	1.705 (1.514–1.921) *p* < 0.001	1.535 (1.314–1.793) *p* < 0.001
Age	1.040 (1.020–1.060) *p* = 0.001	1.023 (0.997–1.049) *p* 0.074
Female sex	0.900 (0.660–1.120) *p* = 0.472	0.580 (0.411–0.818) *p* = 0.002
PU presence	2.507 (1.841–3.414) *p* < 0.001	1.500 (1.059–2.126) *p* = 0.020
Concordance (Harrell)		0.708

*PU* Pressure Ulcers, *IADL* Instrumental Activities of Daily Living, *ACB* Anti-Cholinergic Burden, *SPMSQ* Short Portable Mental Status Questionnaire, *CFS* Clinical Frailty Scale, *CIRS* Cumulative Illness Rating Scale, *MNA* Mini Nutritional Assessment

## Discussion

Our study pointed out the critical relationship between PU presence, frailty, and long-term mortality in hospitalized older adults and, to the best of our knowledge, originally demonstrated the distinct contribution of frailty to increased mortality.

In particular, our findings showed that each increment in CFS score was associated with a 53% increased risk of mortality and the presence of PU carries an additional 50% increased risk of mortality at 18 months based on the same level of frailty, independent of age, sex, and multimorbidity. Such adjustments are required because, even though frailty represents the depletion of physiological reserves, and evidence shows that a substantial proportion of frail individuals are multimorbid, yet a much smaller proportion of those with multimorbidity are frail, indicating that while multimorbidity can contribute to frailty, the reverse is less frequent. Additionally, frailty typically develops later in life, whereas multimorbidity may develop earlier, suggesting a temporal disparity in their onset [28].

So far, the role of PUs as accelerators of mortality has been documented in older adults, acting synergistically with geriatric syndromes (such as hypokinetic syndrome, urinary incontinence, and falls) in worsening the individual’s frailty trajectory. Jaul and Calderon-Margalit, for instance, showed that factors such as low BMI, anemia, dementia, and severe disability contribute to both ulcer development and increased mortality [29]. On the other hand, solid evidence [13, 30] found that clinical frailty (as measured by CFS) is associated with mortality. In particular, Rueegg et al. have shown an association between a one-point worsening of CFS scores and survival, emphasizing the value of considering frailty as a continuous spectrum rather than a binary classification [31] to encompass the complexity and the individualized clinical trajectories of frailty. However, only scant evidence has investigated the contribution of frailty in mediating PU risk [14, 32] and the association between PU and long-term mortality in very old age patients [7]. This is, to our knowledge, the first study to combine the evaluation of PU with the use of the CFS as a continuous variable for prognostic purposes, offering a novel and comprehensive perspective on their interplay in predicting clinical outcomes.

The association between frailty and PU may be explained by overlapping systemic underpinnings. Frailty is often accompanied by sarcopenia, weight loss, reduced mobility, and impaired nutrition, all of which create a physiological environment that predisposes individuals to impaired skin resilience and delayed repair [33]. PU may thus be conceptualized as a localized manifestation of frailty, reflecting diminished tolerance to stressors such as immobility and shear. Beyond local tissue damage, on the other hand, PU appearance marks the final common pathway that accumulates and progresses over the course of years as a result of the synergistical action of geriatric syndromes [29]. Social factors, including isolation and limited engagement in care, may further exacerbate both conditions, reinforcing their combined prognostic significance [34]. To our understanding, frailty acts as a permissive substrate that sustains the presence of PU based on the conceptual framework of clinical deficit accumulation for frailty [35]. As a result, ‘*skin failure’* would catalyze frailty towards a *failure to thrive*, collapsing all the individual’s functional reserve until death.

The lack of significance of the interaction term between CFS and PU presence may be attributed to the advanced level of frailty within our sample, which is likely to be influenced by the specific demographic characteristics of the Liguria region [36]. Such advanced frailty may have created a *ceiling effect* that hampered the interaction and, therefore, could be reversed by studies on a broader sample size.

One strength of our study lies in the robust frailty stratification, which integrates the assessment of PU using validated assessment tools. Furthermore, the inclusion of a representative cohort of oldest-old individuals from a geriatric unit enhances the applicability of our findings to real-world clinical practice.

However, it is important to acknowledge certain limitations of our study. Firstly, our reliance on data from a single hospital may limit the generalizability of the results. Secondly, although all assessments were conducted by trained geriatricians and nurses, some degree of measurement bias cannot be excluded, particularly for clinical evaluations such as pressure ulcer staging. Thirdly, despite adjustment for key confounders, residual confounding from unmeasured variables (e.g., socioeconomic status, lifestyle factors) remains possible. Moreover, BMI, although collected for MNA assessment, was not digitized in our dataset and therefore could not be included in the analyses. Additionally, further research conducted in diverse settings and with larger sample sizes could help to build upon our findings and refine our understanding.

In conclusion, our study originally underscored the independent prognostic significance of both PU and frailty, regardless of multimorbidity. A multidimensional approach to old-age patients, that considers both frailty and PU, is essential for accurate long-term prognostication in hospitalized old-age individuals and may as well ultimately reduce frailty risk or progression and the development of PU.

## Supplementary Information

Below is the link to the electronic supplementary material.

Supplementary file1 (DOCX 17 KB)
