# Supplementary material for: Impact of pressure ulcers and frailty on long-term mortality: a prospective cohort study of hospitalized older adults
Source: Eur Geriatr Med. 2025 Nov 14;17(2):665–72. doi: 10.1007/s41999-025-01331-8 (PMC13109228; doi:10.1007/s41999-025-01331-8)
Supplement: Supplementary file 1 — Supplementary file1 (DOCX 17 KB) [file 41999_2025_1331_MOESM1_ESM.docx]

**Supplementary Materials**

**S1** Clinical phenotype of the study population as for frailty status

|  | CFS <5  (N=59, 18%) | CFS ≥5  (N=265, 82%) | *p* |
| --- | --- | --- | --- |
| Male sex, num (%) | 33 (56) | 94 (35) | 0.003 |
| Age, median (IQR) | 81 (11) | 87 (9) | <0.001 |
| Triceps Skin Fold, median (IQR) | 17 (11) | 16 (11) | 0.094 |
| Mid-Arm Circumference, median (IQR) | 27 (4.75) | 24 (5) | <0.001 |
| Mid-Thigh Circumference, median (IQR) | 32.5 (5.25) | 29.5 (5.5) | <0.001 |
| Hand Grip, median (IQR) | 11.1 (5.5) | 6.9 (6.25) | <0.001 |
| Conley’s scale, median (IQR) | 2 (3) | 5 (3) | <0.001 |
| IADL, median (IQR) | 6 (5) | 1 (3) | <0.001 |
| Barthel Index, median (IQR) | 90 (45) | 25 (45) | <0.001 |
| CIRS-comorbidity index, median (IQR) | 5 (2) | 5 (3) | 0.210 |
| CIRS-severity index, median (IQR) | 2.19 (0.46) | 2.23 (0.71) | 0.222 |
| SPMSQ, median (IQR) | 1 (6) | 6 (3) | <0.001 |
| MNA, median (IQR) | 24.5 (5) | 15.5 (9.5) | <0.001 |
| ACB score, median (IQR) | 1 (1) | 1 (2) | <0.001 |
| N° medication pre-hospitalization, median (IQR) | 6 (4) | 6 (4) | 0.280 |
| 4AT, median (IQR) | 0 (1) | 4 (7) | <0.001 |
| PU presence, num (%) | 6 (10) | 146 (55) | <0.001 |
